# CROSS-NATIONAL STUDIES OF RISK FACTORS FOR COGNITIVE DECLINE AND DEMENTIA

**DOI:** 10.1093/geroni/igae098.2199

**Published:** 2024-12-31

**Authors:** Andrew Steptoe, Laura Brocklebank

**Affiliations:** University College London, London, England, United Kingdom; University of Oxford, Oxford, England, United Kingdom

## Abstract

Cross-national studies of cognitive decline can provide insights into the relevance of risk factors in different cultural settings. They help determine whether relationships are universal or depend on the lifestyles and social context prevailing in different countries. However, cross-national studies present challenges. One important issue is harmonization of self-report and cognitive measures, and this has been greatly facilitated by the Gateway to Global Aging initiative. But there are other factors related to consistency in data collection that require careful consideration. These will be illustrated by a study relating physical activity and sleep patterns to cognitive decline and dementia risk in England using ELSA (the English Longitudinal Study of Ageing) and in China using CHARLS (The China Health and Retirement Longitudinal Study). Absolute levels of physical activity and sleep vary greatly among older people living in urban and rural settings, and with histories of predominantly non-manual work (ELSA) and more mixed occupations (CHARLS). These factors color self-report measures, making comparison difficult. In this study, we have been carrying out 8-day wrist-worn accelerometry in ELSA and CHARLS to provide objective data on movement behaviours (physical activity, sedentary behaviour, and sleep) in more than 20,000 individuals. This presentation highlights challenges related to consistency of data collection, variations in instrumentation, differences in response and adherence rates, and the suitability of machine-learning behavior classification models developed in one cohort but applied in very different people and/or contexts. We will describe our approaches to resolving these issues in order to carry out robust cross-national comparisons.

